# Ultrabroadband near-infrared emission profiling in multicore optical fibers doped with Er^3+^ and Yb^3+^/Tm^3+^/Ho^3+^ ions

**DOI:** 10.1038/s41598-025-88411-8

**Published:** 2025-02-04

**Authors:** Jakub Markiewicz, Marcin Kochanowicz, Tomasz Ragiń, Piotr Miluski, Wojciech A. Pisarski, Joanna Pisarska, Marta Kuwik, Dominik Dorosz, Martin Leich, Matthias Jäger

**Affiliations:** 1 Faculty of Electrical Engineering, Bialystok University of Technology, 45D Wiejska Street, 15-351 Bialystok, Poland; 2 Institute of Chemistry, University of Silesia, 9 Szkolna Street, 40-007 Katowice, Poland; 3 Faculty of Materials Science and Ceramics, AGH University of Science and Technology, 30 Mickiewicza Av, 30-059 Krakow, Poland; 4 Leibniz Institute of Photonic Technology, Albert-Einstein-Str. 9, 07745 Jena, Germany

**Keywords:** Lasers, LEDs and light sources, Lasers, LEDs and light sources

## Abstract

This paper presents novel multicore fibers with broadband emission in the 1.4–2.1 μm range. Based on our previous work with barium gallo-germanate glasses and fibers, the 4 and 11-core fiber structures, each containing core glasses doped with Er^3+^ and Yb^3+^/Tm^3+^/Ho^3+,^ were developed. Our efforts have resulted in flat emission in 3 dB and 10 dB bands, achievable under excitation at 796 nm, 808 nm, 940 nm, and 980 nm. The measured emission spectra of these fibers are described as a superposition of emission bands from each individual core, corresponding to the transitions of Er^3+^, Tm^3+^, and Ho^3+^ ions. Our method demonstrates the potential of emission profiling in multicore optical fibers as a new way to construct eye-safe broadband fiber sources.

## Introduction

Fiber optics technology can rapidly develop due to its wide application in many fields. Actually, much research focuses on near-infrared emission (NIR) due to it is application in telecommunications (II and III window) medicine, gas sensing, and broadband amplified spontaneous emission (ASE) sources^1–3^. Near-infrared emission in active glasses and fibers can be easily obtained by doping active material with rare-earths, transition metals such as nickel, and quantum dots^4–9^. The doping of glasses and nano-crystals with several elements raises a problem in optimizing the concentration of dopants^1,10–12^. While dopants in the form of ytterbium, erbium, or thulium are easily pumped with commercially available semiconductor sources operating at the wavelength of around 800 nm and 980 nm, there is a difficulty with direct pumping of holmium ions^13^. An efficient way of obtaining emission for this element is to use energy transfer between donors such as Tm^3+^, Yb^3+,^ and acceptor in the form of Ho^3+^ ions^10,14–18^. The use of energy transfer to obtain broadband emission in optical fibers raises a problem in optimizing the concentration of active dopants and using a glass matrix that allows fiber drawing and has low phonon energy. Optical fibers fabricated by the MCVD method suffer high phonon energy, reaching a value around 1100 cm^−1^_,_ and dopant concentration quenching, negatively affecting energy transfer efficiency. Strong and highly covalent Si-O bonds lead to multi-phonon relaxation and further energy wasting in the form of heat^19,20^. On the other hand, these fibers still offer low losses and good mechanical and thermal properties, which can be crucial for application in fiber lasers. Heavy metal oxide (HMO) glasses have relatively low phonon energy (~ 700 cm^−1^) compared to the previously mentioned silica, but their mechanical and thermal properties are poor, and some of these glasses, such as lead glasses, can be toxic^21–26^. As an excellent compromise between silica and HMO glasses, germanate, and antimony glasses can be considered due to their phonon energy reaching a value of about^27,28^ 800 cm^−1^. This value allows us to achieve high energy transfer efficiency and doping concentrations while we consider doping with multiple elements such as erbium, thulium, and holmium^29^. Due to low losses, germanate glasses have good optical properties for optical fiber applications, reaching even 0.25 dB/m after fabrication with rod-in-tube method^13,30–32^. As mentioned earlier, the broadband emission in optical fibers can be profiled by changing the concentration of dopants and selecting appropriate glass matrices. However, it is also worth considering extending these methods to include multicore design^33^.

The design of multicore fibers doped with rare-earth ions opens up new possibilities for advanced ASE sources. A key advantage is that multiple cores, each potentially doped with different rare-earth ions, allow for a broader emission spectrum due to the superposition of luminescence bands from various ions. Additionally, MCFs have a higher volume of RE-doped glass in fiber, which is proportional to the number of cores, which can enhance the output power of ASE sources. The multicore structure also reduces the fiber length required to absorb pump radiation, resulting in higher output power than conventional, single-core, double-clad fibers. So far, constructions of active multicore optical fibers have been presented in the literature; however, these solutions focus on fibers in which all cores are doped with in the same way (e.g., Er^3+^ or Er^3+^/Yb^3+^)^34–36^. Currently, there is a lack of solutions based on the use of multicore designs with different active dopants aimed at achieving broadband emission in the near-infrared range. Additionally, research on the impact of multicore structures on obtaining broadband emission in the mentioned range is also missing. Changing the structure, size, and dopant concentrations of individual cores makes it possible to profile emission bands, which can be defined as a superposition of emission bands from individual cores. This approach has another important advantage, which involves an increase in the volume of active dopant glass per meter of fiber, which is proportional to the number of active cores in optical fiber^37–39^. This paper explores the development of novel multicore optical fibers doped with rare-earth ions designed to achieve ultrabroadband near-infrared emission. By leveraging unique glass compositions and multicore structures, we demonstrate a significant enhancement in emission properties, making these fibers promising candidates for applications in telecommunications, medical diagnostics, and broadband ASE sources. Moreover, by modifying the structure (the number of cores, size, and positioning), it is possible to profile the emission spectrum (3dB and 10dB bandwidth) and adjust the pump power distribution within the fiber.

## Methods

Barium gallo-germanate (BGG) core glasses with a composition (mol%): 48.85GeO_2_-25Ga_2_O_3_-10BaO-15Na_2_O-0.5Yb_2_O_3_-0.4Tm_2_O_3_-0.05Ho_2_O_3_ and 49.8GeO_2_-25Ga_2_O_3_-10BaO-15Na_2_O-0.2Er_2_O_3_ were prepared by classic melting and quenching method in electric furnace (CZYLOK Company, Jastrzębie Zdrój, Poland). Set of high-purity materials (99.99%, Sigma-Aldrich, Saint Louis, MO, USA) was melted in a platinum crucible at the temperature of T = 1500 °C for 30 min. The molten glass was poured into a stainless steel form and annealed at 600 °C for 12 h. Absorption properties were measured using Stellarnet Green-Wave spectrometer ranging from 300 nm to 900 nm. Near-infrared luminescence properties of glass samples were measured under excitation of high-power LIMO laser diodes (796, 808, 940 and 980 nm, P_opt_ = 1–30 W) with Acton 2300i monochromator. To prepare the 11-core fiber preform, 10 holes were drilled in a ring shape with an additional hole in the center of the inner cladding. Rods were put to the drilled structure—10 glass rods doped with Yb^3+^/Tm^3+^/Ho^3+^ were placed around the Er^3+^ doped core rod. The structure was placed in a glass tube (outer cladding). In the case of the 4-core preform, 3 outer holes were drilled around the hole in the center of the preform. The same described glass was used in the outer structure (Yb^3+^/Tm^3+^/Ho^3+^ doped glass) and center of the preform (Er^3+^ doped glass). Inner cladding with cores was put in a glass tube. The proposed 11-core and 4-core fiber designs are a direct evolution of our previous work on dual-core fibers, where the near-infrared emission band was insufficiently broad, and there was a pronounced disparity in emission intensity, particularly with an overly dominant peak from erbium ions^38^. To address these limitations, we developed new multicore structures that focus on approximately a tenfold (11-core) and twentyfold (4-core) increase in the in the volume of active (RE-doped) core glass per meter of fiber compared to standard single core-fibers. The 11-core design features smaller outer core diameters compared to the 4-core design, optimizing the emission profile and enhancing the overall intensity and flatness of the emission spectrum. Refractive indexes of core, inner cladding, and outer cladding were 1.74, 1.62 and 1.51, respectively. Fiber was drawn from 10 cm preform at the temperature of 980–1000 °C and coated with a polymeric layer. Spectroscopic properties of the optical fiber and core glasses have been measured in the range of 1300–2300 nm with Yokogawa AQ6375B optical spectrum analyzer under pumping with high-power LIMO diodes operating at the wavelengths of 796, 808, 940 and 980 nm. Measurements of ASE power were carried out with Thorlabs PM16-401 thermal detector. Excited state lifetime in optical fiber was measured under pumping by a 980 nm fiber-coupled single-mode diode at different pump powers. The fluorescence was excited by 1.5 ms pump pulses. The fluorescence signal of the pumped fiber was transversally collected by a 400 μm passive fiber and measured with an InGaAs detector for Er^3+^ and an extended InGaAs detector for Tm^3+^ and Ho^3+^ emission, respectively using a 1 GHz oscilloscope (Keysight model DSOX6004A).The numerical analysis of optical properties for the proposed optical fiber design was performed using the mode solver of RP Fibre Power software. The ASE was simulated at single-end fiber excitation. Pump power absorption in fibers was simulated in TracePro software.

## Results and discussion

### Spectroscopic properties of core glasses

**Fig. 1:**
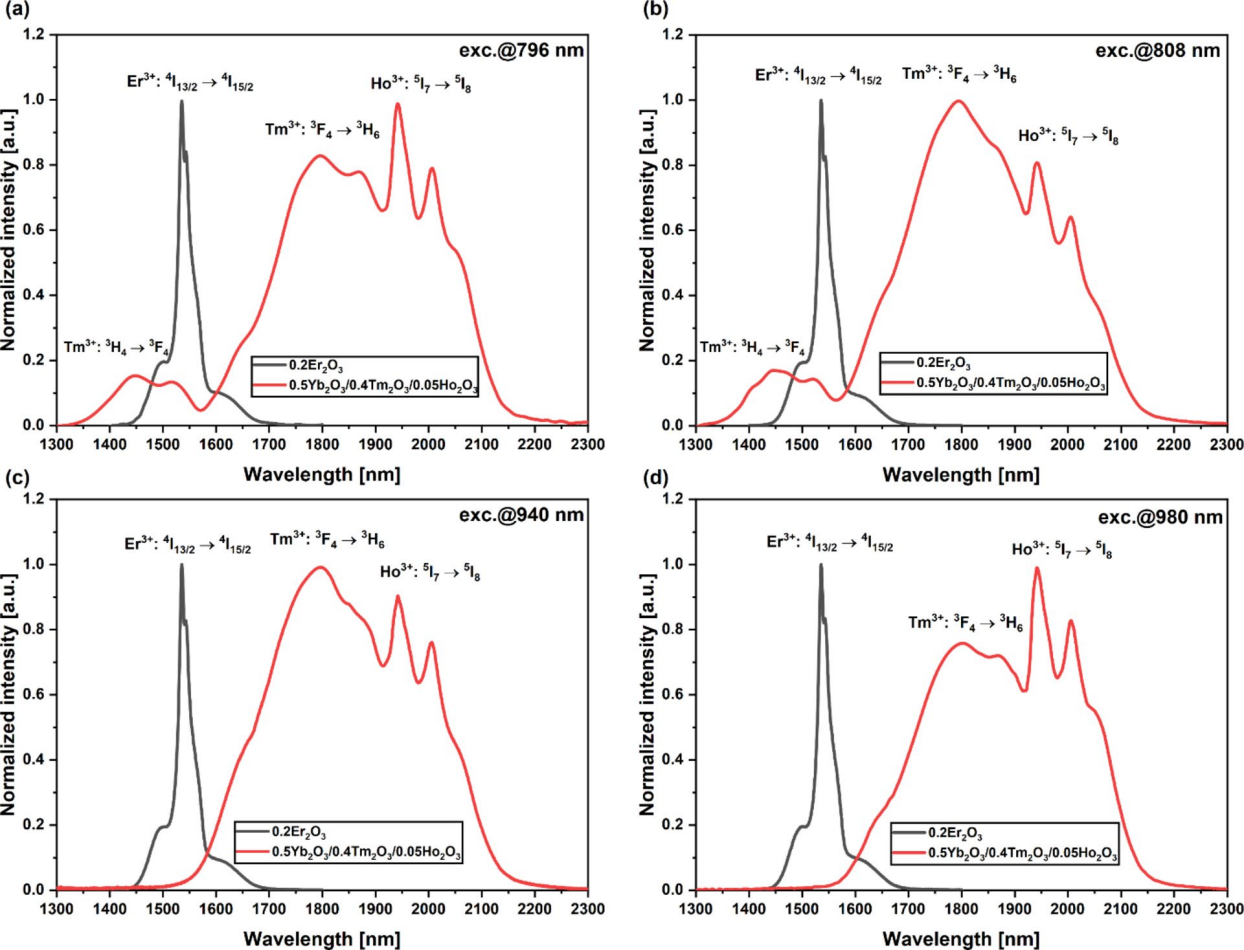
Luminescence spectra of fabricated BGG glass under excitation at (**a**) 796 nm (**b**) 808 nm (**c**) 940 nm (**d**) 980 nm.

Absorbance spectra of both samples doped with Er^3+^ and Yb^3+^/Tm^3+^/Ho^3+^ ions is presented in our previous work on the dual-core optical fiber^38^. To investigate the possibility of obtaining broadband near-infrared emission fully, glass samples were examined under excitation with different pump wavelengths. Figure 1 presents the luminescence spectra for four pumping variants for both glass samples.

In the case of the glass sample doped with Er^3+^ for all four investigated pumps, we obtained luminescence at 1535 nm, which can be attributed to the ^4^I_13/2_ → ^4^I_15/2_ transition of erbium ions. However, it is worth mentioning that emission efficiency is related to the pump wavelength and absorption band, which we are using to obtain NIR emission at 1535 nm. For the second glass sample doped with Yb^3+^/Tm^3+^/Ho^3+^ ions in case of pumping at 796 nm, we obtained broad emission as a result of energy transfer between thulium and holmium ions. In this case, Yb^3+^ ions don’t participate as a donor for Tm^3+^ and Ho^3+^ ions due to the lack of the absorption band at 796 nm for Yb^3+^ ions. The luminescence band at around 1800 nm is related to the^3^F_4_ →^3^H_6_ transition of Tm^3+^ ions, while peaks at around 2000 nm can be assigned to the^5^I_6_ →^5^I_8_ transition of Ho^3+^ ions. Moreover, it is worth mentioning that there is the presence of a luminescence band at 1420 nm related to the cross-relaxation of Tm^3+^ ions and the transition from a higher^3^H_4_ level to a lower^3^F_4_ energy level. Cross-relaxation of Tm^3+^ ions can be crucial for broadening the Er^3+^ luminescence band towards shorter wavelengths in fabricated multicore optical fiber. For the sample pumped at 808 nm, again, we can notice the cross-relaxation of Tm^3+^ ions at 1420 nm. In contrast to the previous case, we can notice a higher emission intensity of Tm^3+^ against Ho^3+^ ions. For the glass sample pumped at 940 nm, we observed broadband emission from 1600 nm to 2100 nm, but it is worth mentioning that there is a lack of emission band at 1450 nm. The ytterbium ions using this optical pump play the role of a donor for the thulium and holmium ions. Using a 940 nm optical pump the ratio of thulium ions emission intensity to holmium is closest to 1 for all analyzed cases. In the case of the 980 nm pump, energy transfer from Yb^3+^ ions to Tm^3+^ and Ho^3+^ ions resulted in a broad emission spectrum; however, the emission band at 1450 nm, associated with the cross-relaxation of Tm^3+^ ions, was absent.

**Fig. 2:**
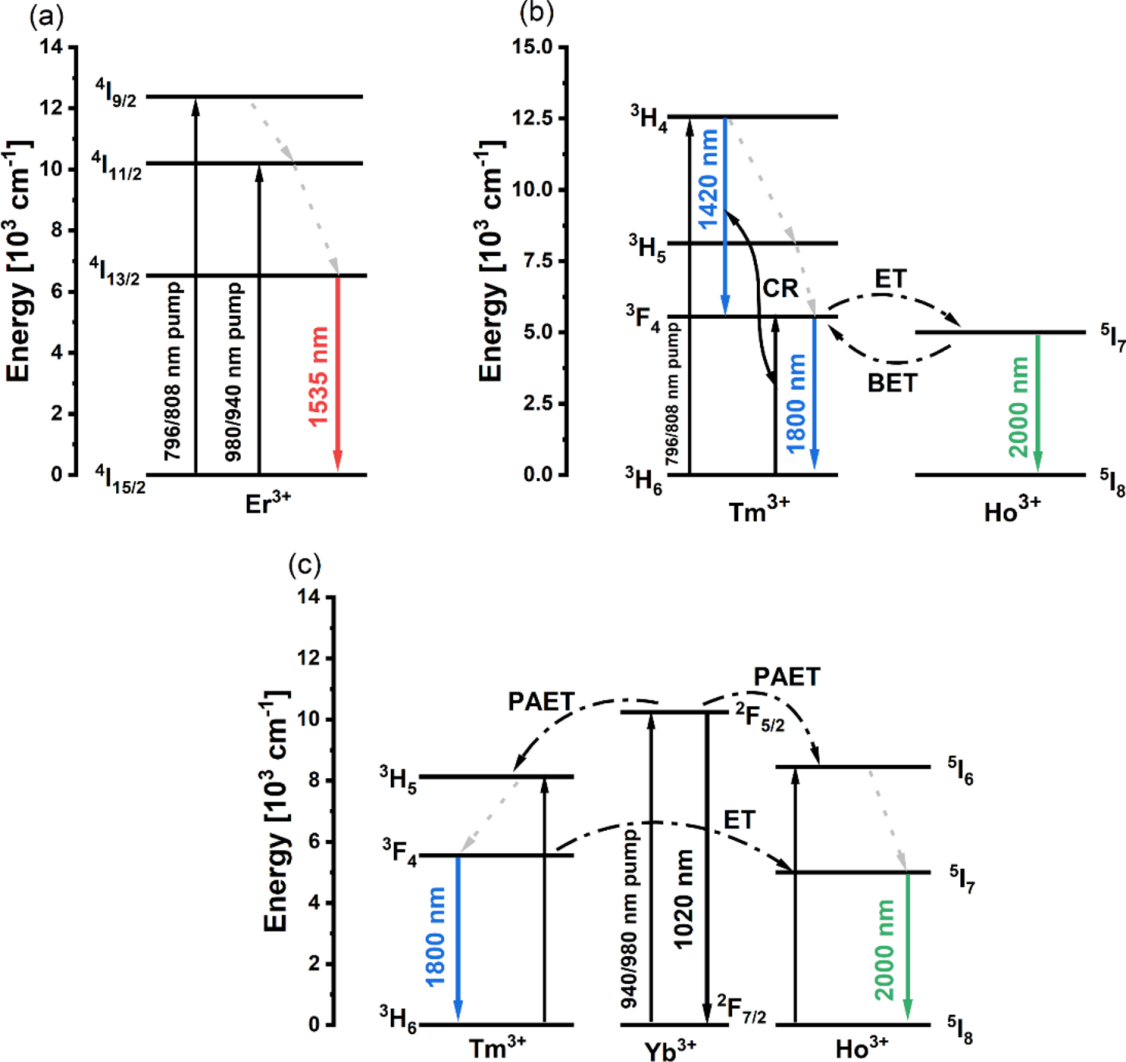
Energy diagrams for (**a**) BGG glass doped with Er^3+^ ions glass under excitation at the wavelength of 796 nm, 808 nm, 940 nm and 980 nm (**b**) BGG glass doped with Yb^3+^/Tm^3+^/Ho^3+^ ions glass under excitation at the wavelength of 796 nm, 808 nm (**c**) BGG glass doped with Yb^3+^/Tm^3+^/Ho^3+^ ions glass under excitation at the wavelength of 940 nm, 980 nm.

Figure 2 presents energy diagrams for both glasses under pumping at different wavelengths. In the case of Er^3+^ (Fig. 2a) doped BGG glass under 980 nm and 940 nm pump excitation, there is the non-radiative transition from ^4^I_11/2_ to ^4^I_13/2_ energy level leading then to the radiative transition from the ^4^I_13/2_ level to the ^4^I_15/2_ ground level. When the 796 nm and 808 nm pumps are used, we have another non-radiative transition from the ^4^I_9/2_ to ^4^I_11/2_ energy level, before the two mentioned transitions. The use of a pump operating at the wavelength of 796 nm and 808 nm for Yb^3+^/Tm^3+^/Ho^3+^ doped glass results in lack of participation of Yb^3+^ ions as donor for Tm^3+^ and Ho^3+^ acceptors. As a result of energy transfer from the^3^F_4_ level of Tm^3+^ ions to the^5^I_7_ level of Ho^3+^ ions, it is possible to obtain emission in the 2 μm band. Direct pumping of Tm^3+^ results in an emission peak at 1.8 μm and another at 1.42 μm which is related to the cross-relaxation of Tm^3+^ ions. It is worth mentioning that these transitions can be quenched for higher concentrations of active dopant, simultaneously resulting in a shift of the emission peak of the Tm^3+^:^3^F_4_ →^3^H_6_ transition towards longer wavelengths^40^. Due to the superposition of Tm^3+^:^3^F_4_ →^3^H_6_ and Ho^3+^:^5^I_7_ →^5^I_8_ transitions, we can observe broad emission in 1.5–2.1 μm range. The last analyzed case is pumping at the wavelength of 940 nm and 980 nm. In this case, we directly pumped ^2^F_5/2_ energy level of Yb^3+^ ions. Next, phonon-assisted energy transfer to Tm^3+^:^3^H_5_ and Ho^3+^:^5^I_6_ levels results in non-radiative transitions to lower levels of thulium and holmium. Due to the presence of 3 active dopants in the glass matrix, there is the possibility of multiple interactions between dopants. Thulium can act as a donor for holmium ions and as an acceptor for ytterbium ions. All interactions between dopants result in broadband emission, which is a superposition of emission bands from Tm^3+^ ions at 1.8 μm and Ho^3+^ ions at 2.0 μm. In the case of using 980 nm and 940 nm pump excitation, there is no possibility of obtaining emission at 1420 nm in the presented glass matrix. Presented luminescence spectra and energy diagrams show that the use of proposed glass matrices for optical fiber allows it to operate at different pump wavelengths. Luminescence decay times and energy transfer efficiencies were presented in our previous works for Er^3+^/Tm^3+^/Ho^3+^ and Yb^3+^/Tm^3+^/Ho^3+^ triply doped glass systems^29,41^. The maximum efficiency of energy transfer (Er^3+^ → Tm^3+^/Ho^3+^) was around 90%, while for the second glass matrix (Yb^3+^ → Tm^3+^/Ho^3+^) obtained efficiency was slightly lower reaching a value of 71.2%.

### Numerical analysis of multicore fibers

**Fig. 3:**
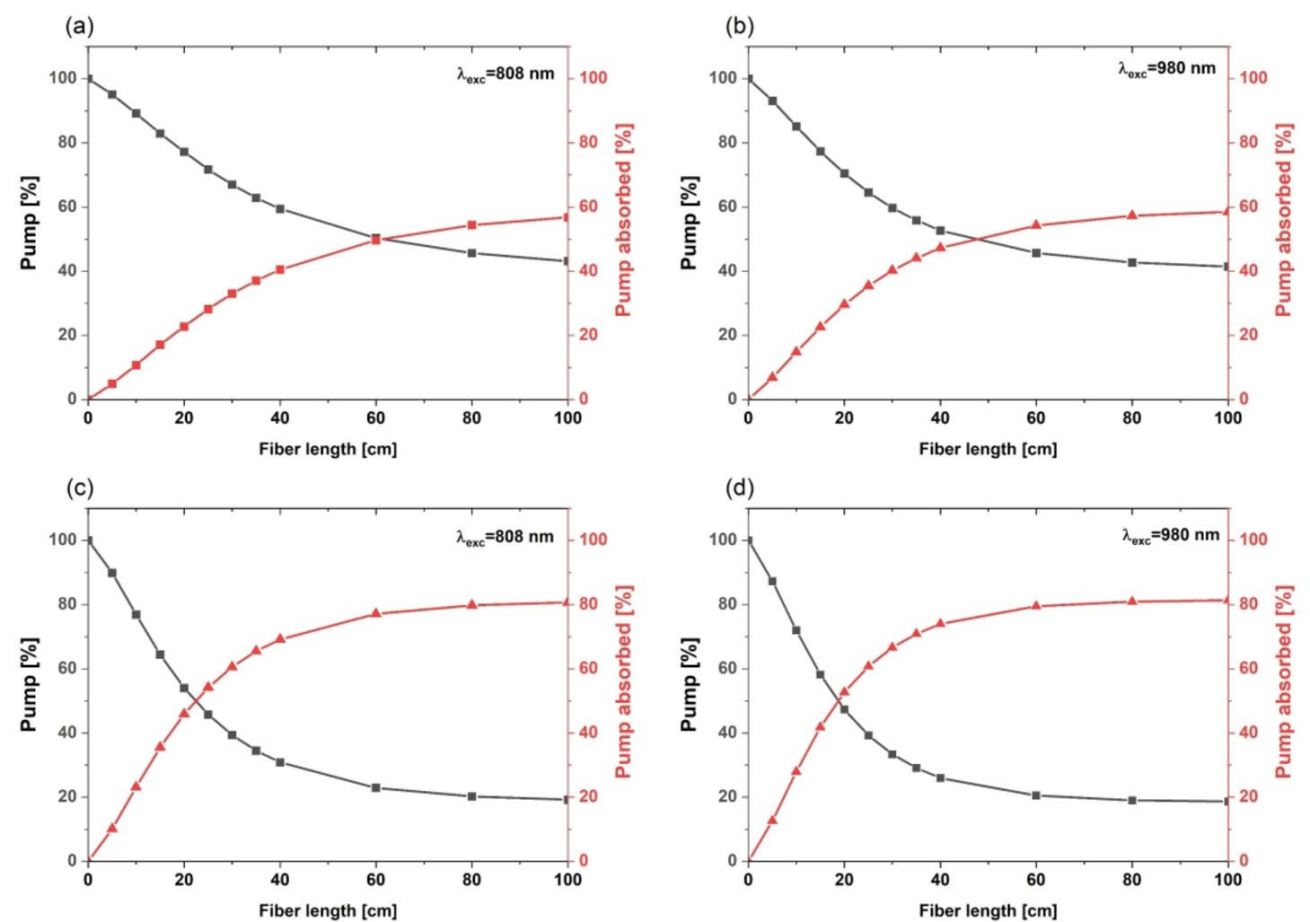
Pump power and absorbed power distribution along 1 m of 4-core fiber under pumping with (**a**) 808 nm, (**b**) 980 nm and for 11-core fiber under pumping with (**c**) 808 nm and (**d**) 980 nm.

Figure 3 presents results for 808 nm and 980 nm pump absorption simulations for 1 m length of 4-core and 11-core fibers. Simulations were performed for absorption coefficients calculated from measured absorbance spectra of presented glasses. Obtained results show that 1 m of 11-core optical fiber absorbs 80% of pump radiation (Fig. 3c, d)).

**Fig. 4:**
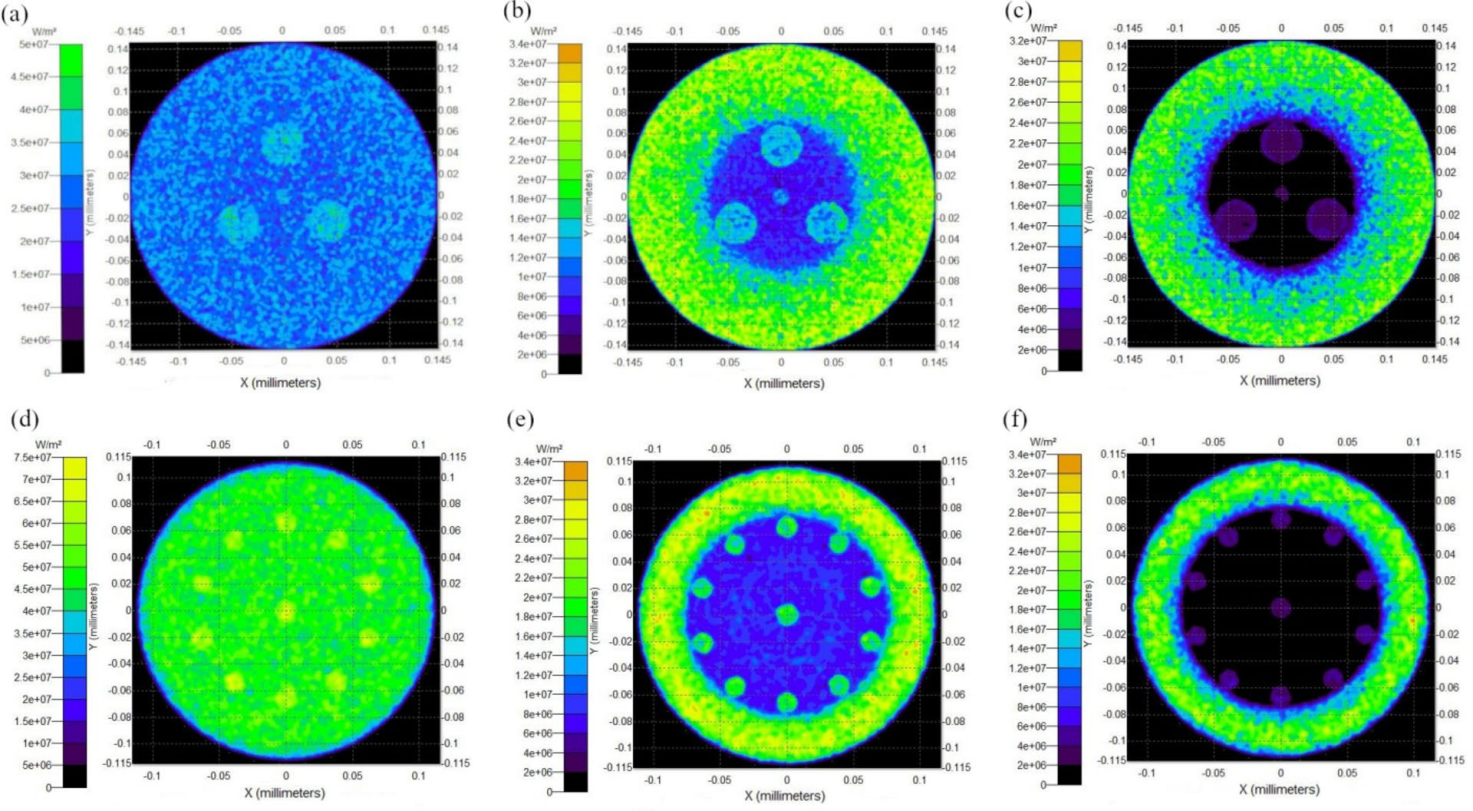
The 808 nm pump power distribution for 4-core fiber at the length of (**a**) 5 cm (Pabs = 4.95%), (**b**) 40 cm (Pabs = 40.56%), (**c**) 100 cm (Pabs = 56.82%) and for 11 core fiber at the length of (**d**) 5 cm (Pabs = 10.11%), (**e**) 40 cm (Pabs = 69.18%), (**f**) 100 cm (Pabs = 80.74%).

Optical fiber containing 4 cores absorbs up to 60% of pump power at 1 m of length, which is 20% less than 11-core structure. In both cases, 980 nm pump radiation was absorbed more than 808 nm due to the strong absorption of Yb^3+^ ions. Figure 4 illustrates simulated pump power (808 nm) distribution across the fiber cross-section at 5 cm, 40 cm, and 100 cm lengths. In both simulated multicore fibers, gradual absorption of pump power in the center of inner cladding and active cores was observed as the fiber length increased. Moreover, it can be observed that the greater the distance of the outer cores from the center of the fiber, the better the absorption of the pump radiation. Proposed multicore optical fibers enable the shorter fiber length required for pump absorption. Standard single, core silica optical fibers require a few meters of fiber. Based on modifying the fiber design, the presented method significantly reduces the required fiber length needed to absorb the optical pump radiation. Moreover, altering the design (core size and positioning) makes it possible to adjust the necessary fiber length depending on different applications.

During the simulation of ASE spectra in optical fibers, pumping at 808 nm and 980 nm was analyzed. For all performed simulations, it was considered that the fiber had no attenuation and that the cores were independent of each other. The reason for this is the high value of Δn between the core and the inner cladding, which causes the radiation to leak into the cladding over a very short distance, significantly smaller than the separation distance between the cores. Numerical modeling of Er^3+^/Tm^3+^/Ho^3+^ doped systems is widely described in literature^42–46^. In the presented model, upconversion and nonlinear processes were not considered. Based on this rate, equations for core doped with Tm^3+^/Ho^3+^ ions are as follows:

1 $$\frac{d{{N}_{3}}_{{H}_{6}}}{dt}=-{W}_{p}{{N}_{3}}_{{H}_{6}}+\frac{{N}_{{3}_{{F}_{4}}}}{{\tau}_{{3}_{{F}_{4}}}}$$

2 $$\frac{d{{N}_{3}}_{{H}_{4}}}{dt}={W}_{p}{{N}_{3}}_{{H}_{6}}-\frac{{N}_{{3}_{{H}_{4}}}}{{\tau}_{{3}_{{H}_{4}}}}$$

3 $$\frac{d{{N}_{3}}_{{F}_{4}}}{dt}={\frac{{N}_{{3}_{{H}_{4}}}}{{\tau}_{{3}_{{H}_{4}}}}-\frac{{N}_{{3}_{{F}_{4}}}}{{\tau}_{{3}_{{F}_{4}}}}-W}_{ET}{{N}_{3}}_{{F}_{4}}{{N}_{5}}_{{I}_{8}}$$

4 $$\frac{d{{N}_{5}}_{{I}_{8}}}{dt}={-W}_{ET}{{N}_{3}}_{{F}_{4}}{{N}_{5}}_{{I}_{8}}+\frac{{N}_{{5}_{{I}_{7}}}}{{\tau}_{{5}_{{I}_{7}}}}$$

5 $$\frac{d{{N}_{5}}_{{I}_{7}}}{dt}={W}_{ET}{{N}_{3}}_{{F}_{4}}{{N}_{5}}_{{I}_{8}}-\frac{{N}_{{5}_{{I}_{7}}}}{{\tau}_{{5}_{{I}_{7}}}}-{W}_{ASE}{{N}_{5}}_{{I}_{7}}$$

where *N* is population of ions in the state described in lower index, *τ* is lifetime of state described in lower index, *W*_*p*_ is pump rate, *W*_*ET*_ is energy transfer rate from Tm^3+^:^3^F_4_ to Ho^3+^:^5^I_8_ level and *W*_ASE_ is rate of ASE depleting the Ho^3+^ excited state. Based on rate equations it is possible to calculate forward ASE power propagation along the fiber which can be described as:

6 $${P}_{ASE}^{}\left(\lambda,z\right)={P}_{ASETm}\left(\lambda,z\right)+{P}_{ASEHo}\left(\lambda,z\right)$$

7 $$\frac{{dP\left(\lambda,z\right)}_{ASETm}}{dz}={g}_{Tm}\left(\lambda,z\right){P}_{ASETm\left(\lambda,z\right)}+{S}_{sp}^{Tm\left(\lambda,z\right)}$$

8 $$\frac{{dP\left(\lambda,z\right)}_{ASEHo}}{dz}={g}_{Ho}\left(\lambda,z\right){P}_{ASEHo\left(\lambda,z\right)}+{S}_{sp}^{Ho\left(\lambda,z\right)}$$

9 $${g}_{Tm}\left(\lambda,z\right)={\varGamma}_{Tm}\left[{\sigma}_{e}^{Tm}\right(\lambda\left){N}_{{}^{3}{F}_{4}}\right(z)-{\sigma}_{e}^{Tm}(\lambda\left){N}_{{}^{3}{F}_{4}}\right(z\left)\right]$$

$${g}_{Ho}\left(\lambda,z\right)={\varGamma}_{Tm}\left[{\sigma}_{e}^{Ho}\right(\lambda\left){N}_{{}^{5}{I}_{7}}\right(z)-{\sigma}_{e}^{Ho}(\lambda\left){N}_{{}^{5}{I}_{8}}\right(z)$$] (10)

11 $${S}_{sp}^{Tm}\left(\lambda,z\right)={h\nu\varGamma}_{Tm}{\sigma}_{e}^{Tm}\left(\lambda\right){N}_{{}^{3}{F}_{4}}\left(z\right)\varDelta{\nu}_{sp}$$

12 $${S}_{sp}^{Ho}\left(\lambda,z\right)={h\nu\varGamma}_{Ho}{\sigma}_{e}^{Ho}\left(\lambda\right){N}_{{}^{5}{I}_{7}}\left(z\right)\varDelta{\nu}_{sp}$$

Going towards Er^3+^ doped core rate and ASE equations are as follows:

13 $$\frac{d{{N}_{4}}_{{I}_{15/2}}}{dt}=-{W}_{p}{{N}_{4}}_{{I}_{15/2}}+\frac{{{N}_{4}}_{{I}_{11/2}}}{{\tau}_{{4}_{{I}_{11/2}}}}+\frac{{{N}_{4}}_{{I}_{13/2}}}{{\tau}_{{4}_{{I}_{13/2}}}}-{W}_{ASE}{{N}_{4}}_{{I}_{13/2}}$$

14 $$\frac{d{{N}_{4}}_{{I}_{11/2}}}{dt}={W}_{p}{{N}_{4}}_{{I}_{15/2}}-\frac{{{N}_{4}}_{{I}_{11/2}}}{{\tau}_{{4}_{{I}_{11/2}}}}-{W}_{NR}{{N}_{4}}_{{I}_{11/2}}$$

15 $$\frac{d{{N}_{4}}_{{I}_{15/2}}}{dt}=\frac{{{N}_{4}}_{{I}_{11/2}}}{{\tau}_{{4}_{{I}_{11/2}}}}+\frac{{{N}_{4}}_{{I}_{13/2}}}{{\tau}_{{4}_{{I}_{13/2}}}}-{W}_{ASE}{{N}_{4}}_{{I}_{13/2}}$$

16 $$\frac{{dP\left(\lambda,z\right)}_{ASEEr}}{dz}={g}_{Er}\left(\lambda,z\right){P}_{ASEEr\left(\lambda,z\right)}+{S}_{sp}^{Er\left(\lambda,z\right)}$$

17 $${g}_{Tm}\left(\lambda,z\right)={\varGamma}_{Er}\left[{\sigma}_{e}^{Er}\right(\lambda\left){N}_{{}^{4}{I}_{13/2}}\right(z)-{\sigma}_{e}^{Er}(\lambda\left){N}_{{}^{4}{I}_{15/2}}\right(z\left)\right]$$

18 $${S}_{sp}^{Er}\left(\lambda,z\right)={h\nu\varGamma}_{Er}{\sigma}_{e}^{Er}\left(\lambda\right){N}_{{}^{4}{I}_{15/2}}\left(z\right)\varDelta{\nu}_{sp}$$

For the Tm^3+^/Ho^3+^-doped core, simulations of the forward ASE spectrum were carried out for various 808 nm pump powers (400–2000 mW), as well as the distribution of pump power and forward ASE along the fiber length. In Tables 1 and 2 below, we present simulation parameters for Tm^3+^/Ho^3+^ and Er^3+^ doped cores.

**Table 1:** Simulation parameters for Tm^3+^/Ho^3+^ doped cores.

Quantity	Symbol	Value	Unit
Thulium concentration	N_Tm_	1.65 × 10^26^	m^−3^
Holmium concentration	N_Ho_	2.06 × 10^25^	m^−3^
Fiber length	L_f_	0.25	m
Core radius	r_c_	7.5	µm
Pumping wavelength	λ_exc_	808	nm
Lifetime of level ^3^F_4_	τ_1_	1922	µs
Lifetime of level ^3^H_4_	τ_2_	11	µs
Lifetime of level ^5^I_7_	τ_3_	3332	µs
Energy transfer coefficient Tm → Ho	W_ET_	5 × 10^−21^	m^3^/s
Maximum absorption cross-section (Tm)	σ_a1_	3.29 × 10^−21^	cm^2^
Maximum emission cross-section (Tm)	σ_e1_	5.28 × 10^–21^	cm^2^
Maximum absorption cross-section (Ho)	σ_a2_	12.89 × 10^−21^	cm^2^
Maximum emission cross-section (Ho)	σ_e2_	14.82 × 10^−21^	cm^2^
Pump absorption coefficient at 808 nm	α_Tm_	0.149	cm^−1^

**Table 2:** Simulation parameters for Er^3+^ doped cores.

Quantity	Symbol	Value	Unit
Thulium concentration	N_Er_	8.37 × 10^25^	m^−3^
Fiber length	L_f_	0.25	m
Core radius	r_c_	7.5	µm
Pumping wavelength	λ_exc_	980	nm
Lifetime of level ^4^I_13/2_	τ_4_	7991	µs
Probability of spontaneous transition ^4^I_11/2_ → ^4^I_15/2_	A_31_	163.19^47^	s^−1^
Probability of spontaneous transition ^4^I_11/2_ → ^4^I_13/2_	A_32_	3.29 × 10^−21^	s^−1^
Maximum emission cross-section (Er)	σ_a3_	5.28 × 10^−21^	cm^2^
Maximum absorption cross-section (Er)	σ_e3_	12.89 × 10^−21^	cm^2^
Pump absorption coefficient at 980 nm	α_Er_	0.91^48^	cm^−1^

**Fig. 5:**
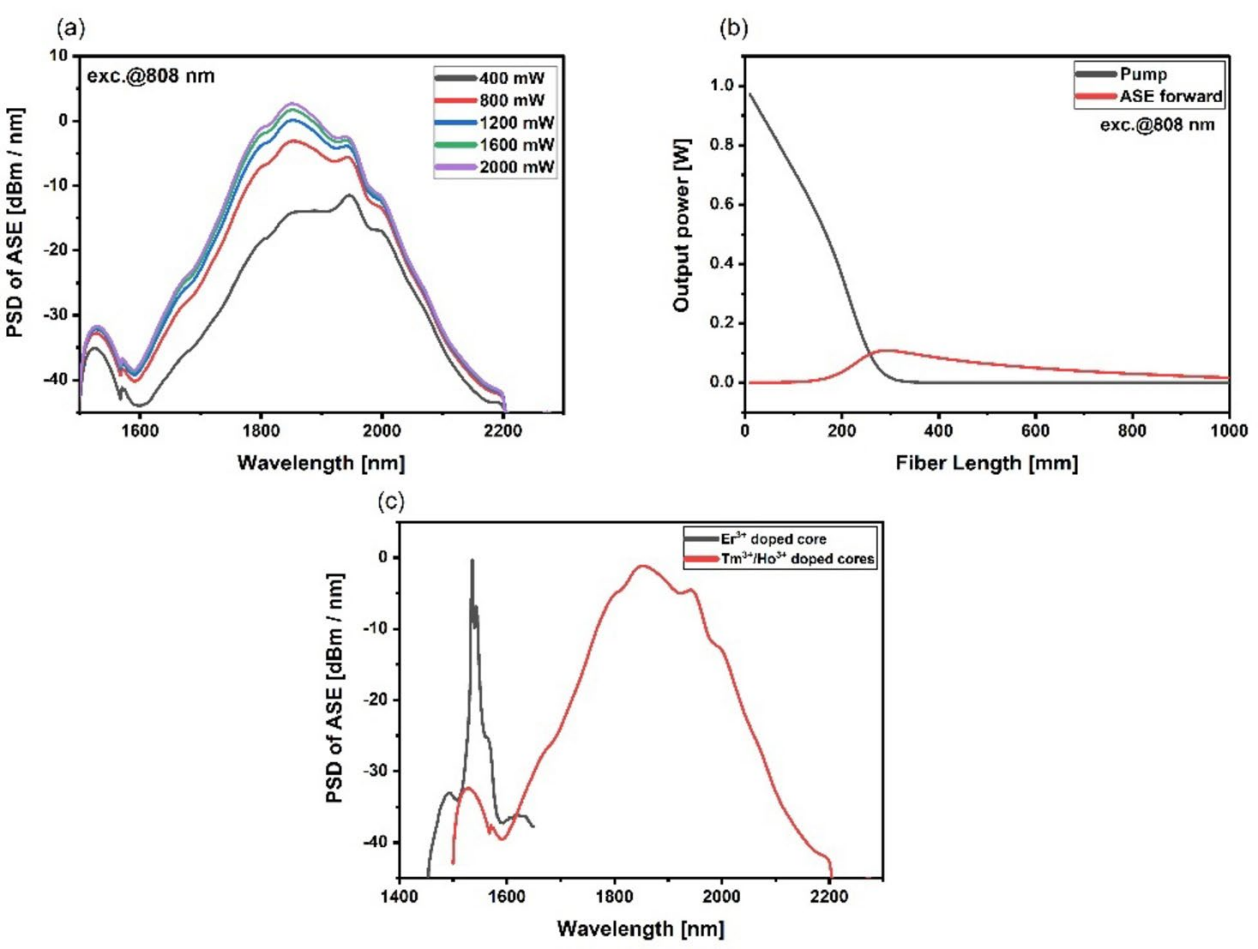
(**a**) Tm^3+^/Ho^3+^ doped core forward ASE spectrum simulations for different pump powers (**b**) Tm^3+^/Ho^3+^ doped core pump and forward ASE distribution along the fiber length (**c**) forward ASE from Er^3+^ and Tm^3+^/Ho^3+^ doped cores.

Results of simulations are presented in Fig. 5. Based on the dependence of pump power as a function of ASE power density, the most significant changes were observed when the optical pump power was increased from 400 mW to 800 mW.

The highest ASE output power density was obtained at a length of approximately 30 cm of the active fiber under pumping with 1 W of optical power. Above 30 cm fiber length strong reabsorption process limits ASE output power. However, the presented results confirm the possibility of obtaining broadband ASE emission as a superposition of emission from particular cores of multicore optical fiber.

### Fabricated multicore fibers

**Fig. 6:**
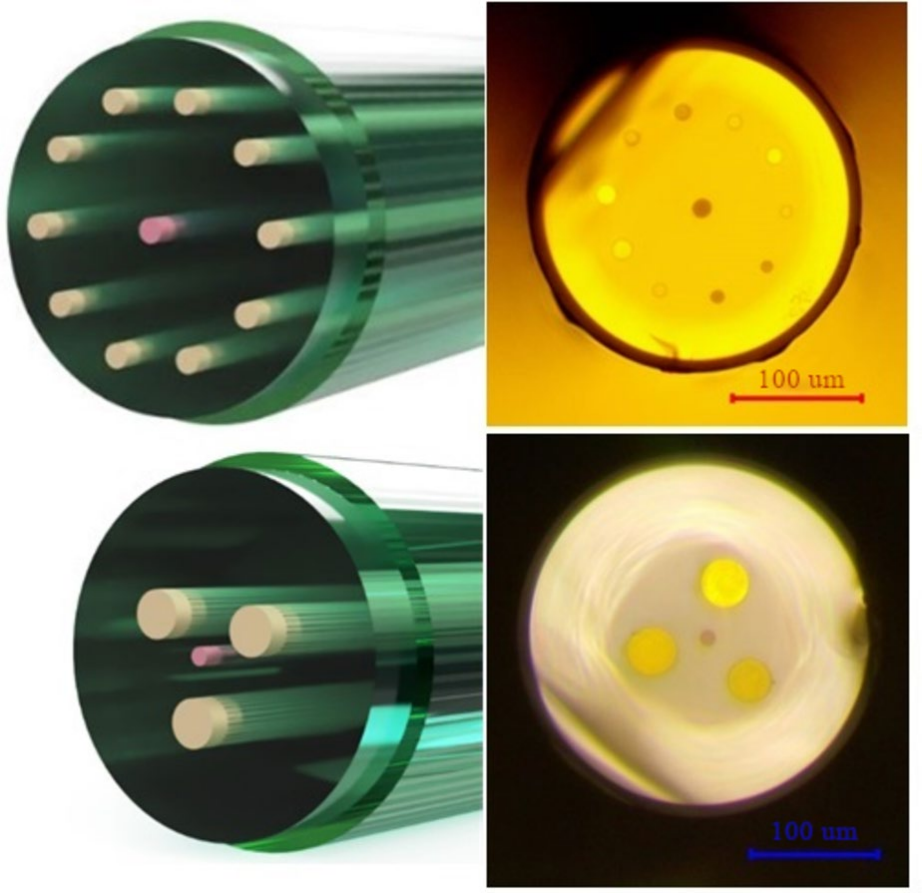
Schemes and cross-sections of drawn fibers.

The presented multicore optical fibers were fabricated using a modified rod-in-tube method. Figure 6 illustrates the preform schemes and cross-section of the drawn fibers. In both fabricated fibers, the central core is doped with Er^3+^ ions, while the outer cores are doped with Yb^3+^/Tm^3+^/Ho^3+^ ions. All measured and calculated parameters of fabricated fibers have been presented in Table 3. Double-clad construction is crucial for pumping multiple cores of the fiber and allows for an increase in pumping efficiency. Gallo-germanate glasses are solid materials suitable for active multicore fibers fabricated using the rod-in-tube method due to their low phonon energy (approximately 800 cm^−1^) and relatively low losses (even 0.25 dB/m)^28,48,49^.

**Table 3:** Properties of fabricated multicore fibers.

Parameter	Symbol	4-core fiber	11-core fiber	Unit
Outer cladding refractive index	n_out_	1.51	1.51	–
Inner cladding refractive index	n_in_	1.62	1.62	–
Core refractive index	n_core_	1.74	1.74	–
Cladding numerical aperture	NA_cladding_	0.59	0.59	–
Core numerical aperture	NA_core_	0.63	0.63	–
Fiber diameter	Φ_fiber_	300	230	µm
Er^3+^ doped central core diameter	Φ_central_	15	15	µm
Yb^3+^/Tm^3+^/Ho^3+^ doped outer cores diameter	Φ_outer_	40	15	µm
Separation between the central and outer core	d_c−o_	21.5	50	µm
Separation between outer cores	d_o−o_	44	25	µm
Total Yb^3+^/Tm^3+^/Ho^3+^ doped cores volume per meter of fiber	V_Yb/Tm/Ho_	3.77	1.77	mm^3^
Er^3+^ doped core volume per meter of fiber	V_Er_	0.178	0.178	mm^3^
Volume ratio (V_Yb/Tm/Ho_/V_Er_)	R	21.33	10	–

### Spectroscopic properties of multicore optical fibers

**Fig. 7:**
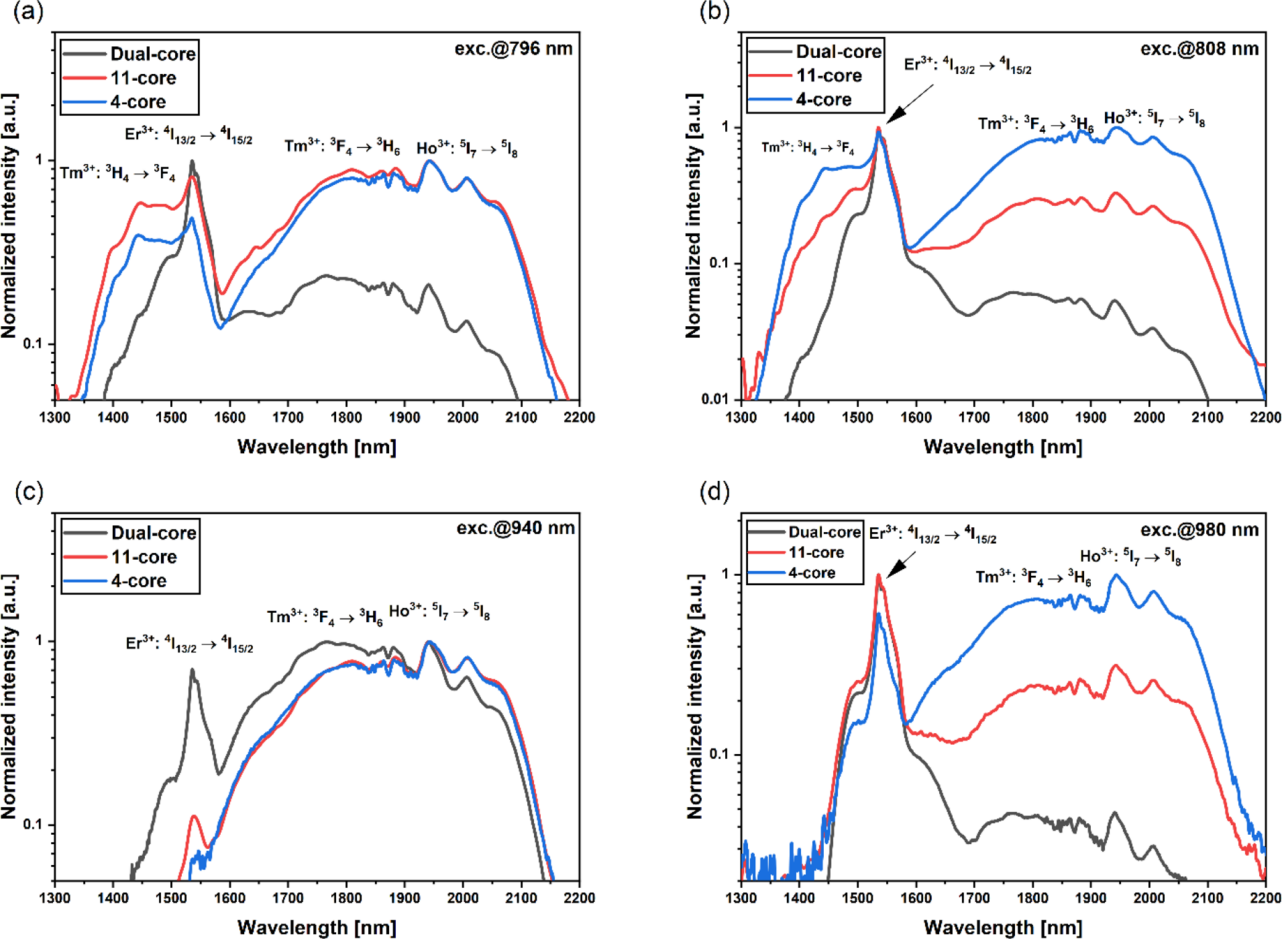
Emission spectra of 1 m length multicore fibers under excitation at: (**a**) 796 nm (**b**) 808 nm (**c**) 940 nm (**d**) 980 nm.

ASE spectra were measured for four different excitation wavelengths under one-end pumping. Spectra were measured for various fiber lengths ranging from 1 to 0.2 m. In all cases, we observed strong near-infrared amplified spontaneous emission spectra as a superposition of emissions from the Er^3+^ doped core and Yb^3+^/Tm^3+^/Ho^3+^ cores. Depending on the pumping variant, we observed green and blue up-conversion, which will be described in the further part of the section for 11-core optical fiber. Figure 7 illustrates the ASE spectra of 1 m length fibers for four pumping variants compared with dual-core optical fiber presented in our previous work^38^. If we look at the luminescence properties of 11-core fiber under 808 nm and 980 nm pumping, a 3dB band is narrow due to the direct the pumping of Er^3+^ ions reaching value of 28 nm. For 796 nm and 940 nm pumps, we obtained a 3 dB band reaching 364 and 360 nm, respectively. This band can be described as a superposition of Tm^3+^ and Ho^3+^ emission bands. The 10 dB band indicates that the broadest emission is achieved with the 796 nm and 808 nm pumps for the 11-core fiber, which can be attributed to the contribution of the cross-relaxation of Tm^3+^ ions at a wavelength of 1420 nm. In case of 940 nm pumping we obtained 543 nm for 10 dB band. It is worth mentioning that the emission intensity of Er^3+^ ions at 1535 nm is lower compared to the luminescence of Tm^3+^ and Ho^3+^ ions. For the 980 nm pump, a broadband emission in a 10 dB band reaching a value of 640 nm was observed. However, there is a lack of emission band corresponding to the^3^H_4_ →^3^F_4_ transition of Tm^3+^ ions, which is crucial for broadening the emission spectrum in presented multicore fibers.

**Fig. 8:**
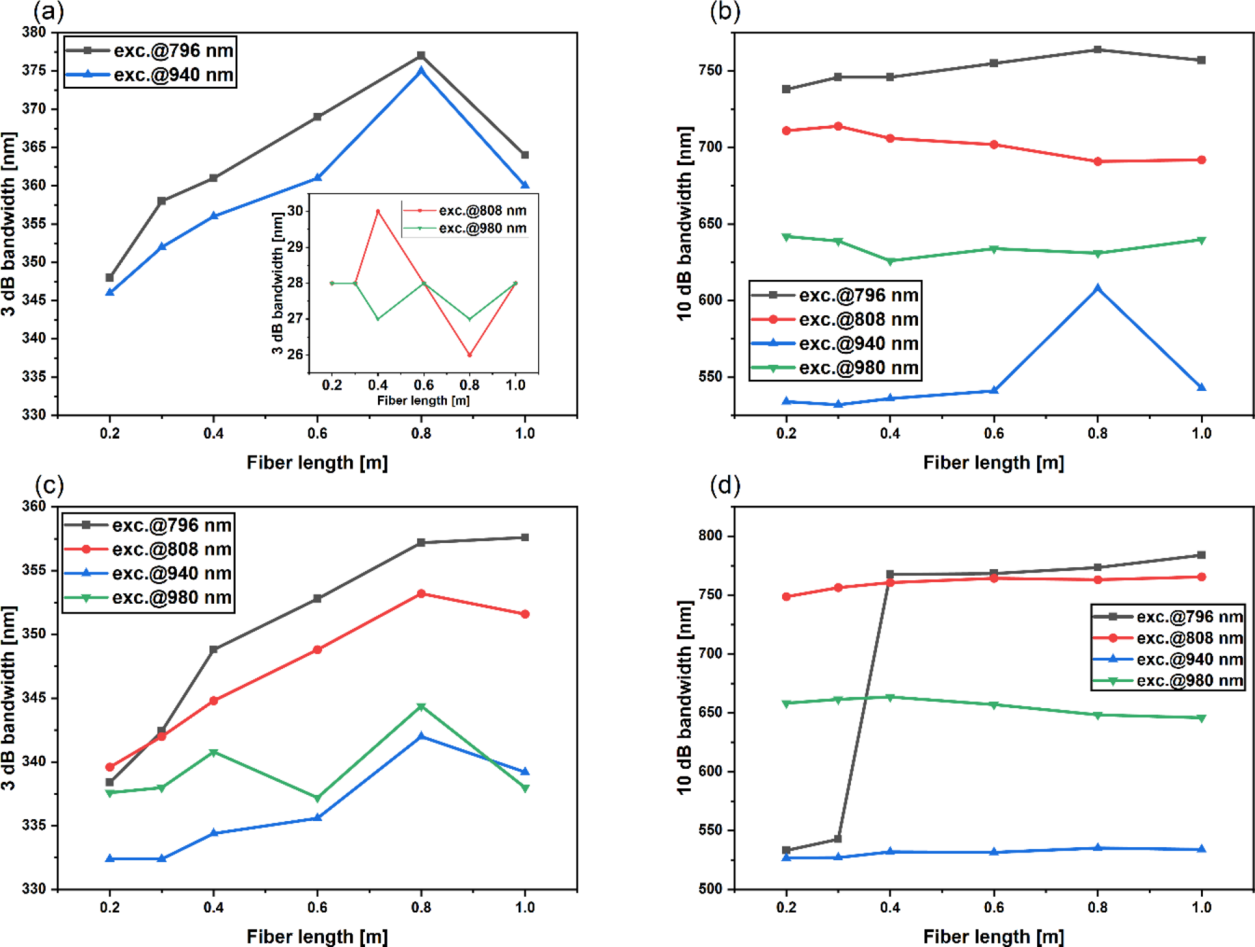
Emission bands of fabricated multicore fibers: (**a**) 3 dB bands of 11-core fiber (**b**) 10 dB bands of 11-core fiber (**c**) 3 dB bands of 4-core fiber (**d**) 10 dB bands of 4-core fiber.

Compared to our previous work on double-core fibers with the same dopant concentrations, the 980 nm pumping variant expanded the 10 dB emission band significantly, increasing it from 155 nm to 640 nm. Analysis of the four-core optical fiber shows that increasing the amount of active material doped with Yb^3+^/Tm^3+^/Ho^3+^ significantly enhances the luminescence intensity in the 1.6 μm to 2.1 μm range under 808 nm and 980 nm pumping. Figure 7a shows that when the 4-core optical fiber was pumped at a wavelength of 796 nm, the emission intensity of Tm^3+^ and Ho^3+^ ions was higher compared to that of Er^3+^ ions, unlike in the 11-core and dual-core optical fibers. The increased emission in the range of 1.6 μm to 2.1 μm results from increasing the amount of Tm^3+^ and Ho^3+^ dopants concentration per meter of fiber. For pumping at 940 nm ASE spectra of 11-core and 4-core optical fibers exhibit nearly identical profiles. Extending emission for 11-core and 4-core optical fibers showcases how altering the fiber’s construction can impact on emission profile rather than optimization of dopant concentrations in single-core optical fibers. Figure 8a shows the influence of fiber length on the 3 dB band. In the 11-core optical fiber, the 3 dB emission band varies with the pumping wavelength, appearing as superposition of Tm^3+^ and Ho^3+^ ions (796 nm and 940 nm pump), and Er^3+^ ions emission band (808 nm and 980 nm pump). As fiber length increases, the 3 dB emission band widens for 796 nm and 940 nm pumps, likely due to the reabsorption of Tm^3+^:^3^F_4_ →^3^H_6_ and^5^I_7_ →^5^I_8_ transitions^42,50^. However, with 808 nm and 980 nm pumps, the stronger Er^3+^ emission negatively affects the flattening of emission spectra in the 11-core fiber. Figure 8b presents the impact of fiber length on 10 dB emission band for four analyzed pumping variants. Pumping at 796 nm promotes ultra-broadband emission in the 1350–2150 nm range, whereas the narrowest emission band was observed with a 940 nm source. For this pump, all cases except 0.8 m fiber showed a 10 dB band as a superposition of Tm^3+^ and Ho^3+^ emission bands. Beyond 0.3 m of fiber length, the emission band narrows under 808 nm pumping, contrasting with other pump variants where 10 dB broadens with the increasing fiber. As mentioned before, the absence of the emission band corresponding to the cross-relaxation of Tm^3+^ ions leads to narrower 10 dB emission spectra. The widest 3 dB band, measuring 377 nm, was achieved by pumping 0.8 m of fiber with a 796 nm laser diode. Correspondingly, the widest 10 dB band, equal to 764 nm, was obtained under the same excitation (796 nm) and fiber length (0.8 m). This 10 dB bandwidth is higher compared to previously reported Er^3+^/Tm^3+^/Ho^3+^ doped double-clad fibers fabricated by the rod-in-tube method (731 nm) and Tm^3+^/Ho^3+^ silica fibers prepared by the MCVD method (645 nm)^29,51,52^.

Based on the results obtained for the 4-core optical fiber (Fig. 8c), the 3 dB emission bandwidth increases with the fiber length up to 0.8 m under excitation at the wavelength of 808 nm and 940 nm. For these two pumping variants beyond 0.8 m, 3 dB band decreases. In the case of pumping at a wavelength of 980 nm, fluctuations in the measurement results can be observed as the fiber length increases (Fig. 8c). If we look at Fig. 8d presenting 10 dB bandwidth as a function of fiber length, we can notice a smaller value change compared to 11-core optical fiber (Fig. 8b).

**Fig. 9:**
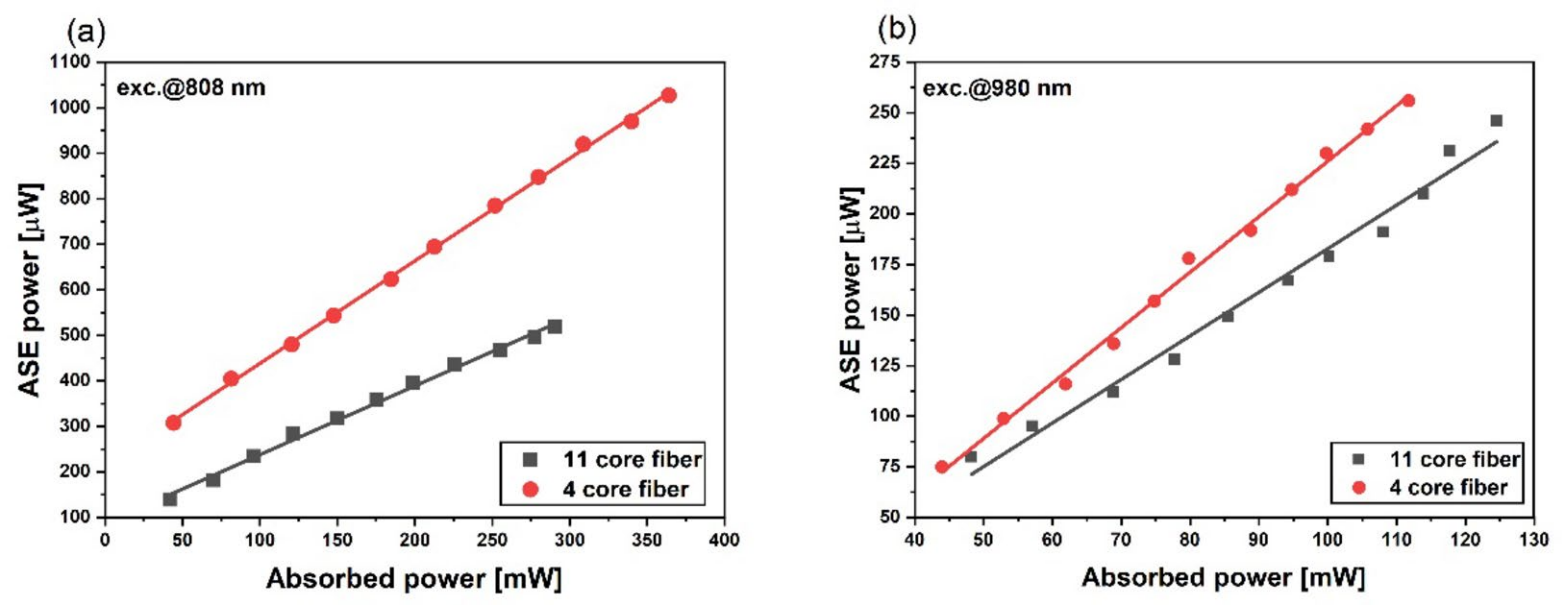
presents measured ASE output power as a function of absorbed pump power.

Figure 9 ASE power of fabricated fibers as a function of absorbed pump power under excitation at (**a**) 808 nm and (**b**) 980 nm.

**Fig. 10:**
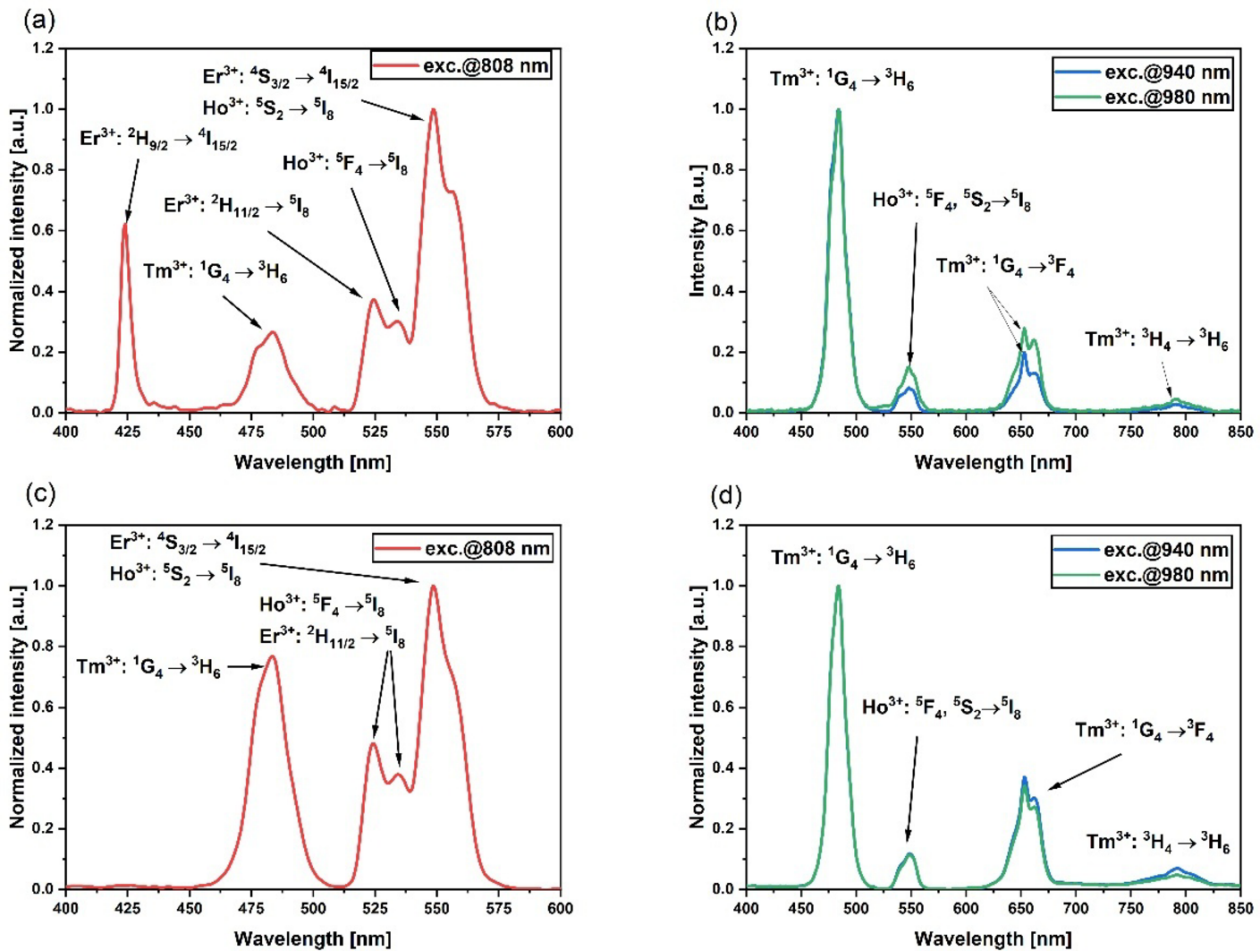
Upconversion spectra of 11-core optical fiber under pumping with (**a**) 808 nm, (**b**) 940 nm, 980 nm laser and 4-core fiber under pumping with (**c**) 808 nm, (**d**) 940 and 980 nm laser.

We noticed higher output power for 4-core fiber in both analyzed pump cases. This is related to a higher amount of active material per meter of fiber and lower losses in the fabricated 4-core optical fiber. We obtained around 1mW of output power for 375 mW of pump power under 808 nm pumping of 4-core optical fiber (Fig. 9a). Under pumping at the wavelength of 980 nm in both presented fibers, we obtained output powers up to 250 µW, which may be due to Tm^3+^ and Ho^3+^ ions being excited only by energy transfer from Yb^3+^ ions. Obtained output power and spectrum with is higher than in silica fiber co-doped with Tm^3+^/Ho^3+^ ions^50^. Weak upconversion emission related to all three rare earths was observed (Fig. 10). When an 11-core fiber is pumped at a wavelength of 940–980 nm, we can observe blue emission at 475 nm. Thulium ions are known for generating blue emission due to the transition^1^G_4_ →^3^H_6_. Red emission at 650 nm with lower intensity was also observed, which can be attributed to the^1^G_4_ →^3^F_4_ transition of Tm^3+^ ions. Pumping fiber at the wavelength of 808 nm favors obtaining green emission. We observed emission bands from Er^3+^ and Ho^3+^ ions at 550 nm, which can also be described as a superposition of emission bands from Er^3+^ and Yb^3+^/Tm^3+^/Ho^3+^ doped cores. Compared to the 11-core optical fiber, the 4-core optical fiber does not exhibit an emission band at a wavelength of 425 nm corresponding to the Er^3+^: ^2^H_9/2_ → ^4^I_15/2_ transition. This absence could be related to differences in power density distribution within the fiber. Reducing the number of cores alters the intensity profile, which may affect the upconversion processes and the resulting emission spectra.

**Fig. 11:**
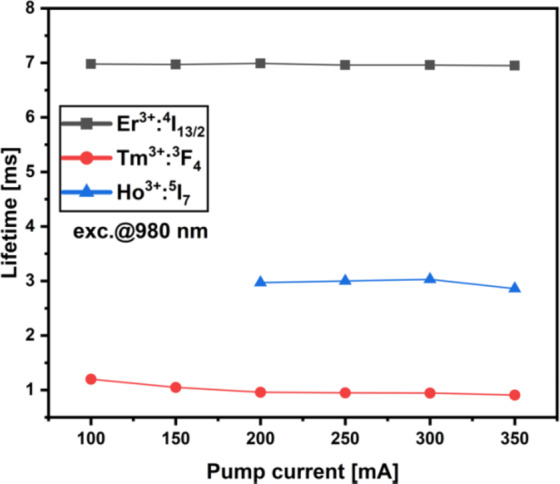
Lifetime of Er^3+^, Tm^3+^ and Ho^3+^ excited levels vs. pump power in 11-core optical fiber.

Figure 11 presents the effect of pump power on the lifetimes of Er^3+^, Tm^3+^, and Ho^3+^ excited levels in 11-core optical fiber. Generally, drawing the fiber from the preform decreased the lifetimes of all analyzed core glasses^53^. A similar effect was shown in silica fiber preforms and drawn optical fibers doped with Yb^3+^, Tm^3+^, and Ho^3+^ where authors suggest that this effect can be mitigated by decreasing the temperature of the heat treatment of preform to a necessary minimum^53^. A minimal decrease in lifetime vs. pump power was observed, which is related to the population of higher RE energy levels and, finally, upconversion emission. The minor lifetime variations observed in Er^3+^, Tm^3+^, and Ho^3+^ excited states suggest reduced ion clustering, which increases the likelihood of radiative transitions within the desired infrared band. This effect is crucial for improving output power within the 1.4–2.1 μm range.

## Conclusions

In conclusion, studying ASE spectra in multicore optical fibers under various excitation wavelengths has provided significant insights into the emission properties of Er^3+^, Tm^3+^, and Ho^3+^ triply-doped optical fibers. The analysis shows that fiber structure and pump wavelength are crucial in profiling the emission spectra. The 4-core and 11-core optical fibers exhibited broad emission bands under 796 nm and 808 nm pumping, with a notable superposition of Er^3+^, Tm^3+^, and Ho^3+^ emission bands. The broadest 3 dB and 10 dB emission bands were achieved at 796 nm excitation, which could be attributed to cross-relaxation processes in Tm^3+^ ions. Comparative analysis with dual-core optical fiber indicates that the increased number of active cores significantly improves ASE flattening and broadens the emission bandwidth. The study of pump power effects on the lifetimes of Er^3+^, Tm^3+^, and Ho^3+^ excited levels in 11-core optical fibers shows that drawing the fiber from the preform generally reduces the lifetimes of all analyzed rare-earth doped core glasses. This trend is consistent with previous observations in silica fibers, where Yb^3+^, Tm^3+^, and Ho^3+^ doping resulted in similar decreases in lifetime. Although this reduction in lifetime is minimal, it is directly correlated to the population of higher rare-earth energy levels, leading to upconversion emission.

Overall, the developed multicore optical fibers present new opportunities for developing broadband ASE sources operating in the 1.5–2.1 μm spectral range.

## Data Availability

The datasets used and/or analysed during the current study available from the corresponding author on reasonable request.
